# Feasibility and Mechanism Analysis of Shenfu Injection in the Treatment of Idiopathic Pulmonary Fibrosis

**DOI:** 10.3389/fphar.2021.670146

**Published:** 2021-07-28

**Authors:** Peipei Liu, Shengnan Yang, Zai Wang, Huaping Dai, Chen Wang

**Affiliations:** ^1^ Department of Pulmonary and Critical Care Medicine, Center of Respiratory Medicine, China-Japan Friendship Hospital, Beijing, China; ^2^ National Center for Respiratory Medicine, Beijing, China; ^3^ Institute of Respiratory Medicine, Chinese Academy of Medical Sciences, Beijing, China; ^4^ National Clinical Research Center for Respiratory Diseases, Beijing, China; ^5^ Chinese Academy of Medical Sciences, Peking Union Medical College, Beijing, China; ^6^ Harbin Medical University, Harbin, China; ^7^ Institute of Clinical Medical Sciences, China-Japan Friendship Hospital, Beijing, China

**Keywords:** idiopathic pulmonary fibrosis, Shenfu injection, network pharmacology, inflammation, apoptosis

## Abstract

**Background:** Idiopathic pulmonary fibrosis (IPF) is disease with high mortality, and its effective treatment is limited. Shenfu injection is a traditional Chinese medicine which can improve circulation and protect cells. In this study, we aimed to investigate the feasibility and mechanism of Shenfu injection in the treatment of IPF.

**Methods:** The components and targets of Shenfu injection were mainly retrieved from the Traditional Chinese Medicine Systems Pharmacology Database and Analysis Platform (TCMSP) database. The targets of Shenfu injection were standardized by UniProt database. The Genecards and OMIM databases was used to search for IPF-related genes. The Venn diagram of gene intersection was drawn using the OmicStudio tools, and the protein-protein interaction network was visualized using the Cytoscape 3.7.2 software. Moreover, the metascape online software was applied to explore the enriched Gene Ontology (GO) terms and Kyoto Encyclopedia of Genes and Genomes (KEGG) pathways, and the Cytoscape 3.7.2 software was used to construct the target-pathway network. Molecular docking was used to visualize the interactions between the main active compounds and targeted proteins. Animal experiments were performed to validate the effects and mechanisms of Shenfu injection.

**Results:** We obtained 46 co-targets of Shenfu injection and IPF. Among the hub target genes, several genes with important functions were enriched, including TNF, IL-6, IL-1B, TP53, JUN, CASP3 and CASP8. The pathway enrichment analysis for the hub target genes identified pathways in infection/inflammation, apoptosis and cancer. Molecular docking results showed that the main active compound Ginsenoside Re had high affinity to the core target proteins. These results suggested that Shenfu injection may have a positive effect in the treatment of IPF. Consistent with this finding, animal experiments showed that Shenfu injection significantly reduced pulmonary fibrosis in a mouse model with inhibition of apoptosis and inflammation by downregulating IL-1β, caspase-3 and phosphorylated NF-κB.

**Conclusion:** Our results demonstrated that Shenfu injection efficiently alleviate bleomycin-induced pulmonary fibrosis through multi-targets in inflammation-, apoptosis- and cancer-related pathways, which provided first evidence and reference to the feasibility of Shenfu injection in the treatment of IPF.

## Introduction

Idiopathic pulmonary fibrosis (IPF) is a progressive fibrosis lung disease with the clinical symptoms of cough and progressive dyspnea. It is common in old men with median age of 65 years. For patients with IPF, the median survival time after diagnosis is between 2 and 4 years (Richeldi et al., 2017). At present, lung transplantation is the only effective treatment for IPF. Pirfenidone and nintedanib are recommended to treat IPF (Behr et al., 2018). However, many patients can hardly afford the burden of lung transplantation and drugs.

Shenfu injection is a traditional Chinese medicine injection that has been approved in 1987 (approval code: SFDA approval number Z20043117, package: 50 ml per bottle) and applied in clinical for 30 years. It is taken from Shenfu decoction and used in clinical acute conditions such as shock, cardiopulmonary resuscitation and heart failure. A prospective randomized controlled study aimed at efficacy and safety found that Shenfu injection can improve 28- and 90-days survival, shorten hospital stays, improve clinical outcomes in patients and has no serious drug-related adverse reactions (Zhang et al., 2017b). Shenfu injection also enhances cellular immunity. Another study in sepsis or septic patients found that Shenfu injection reduced the duration of vasopressor use and ICU stay, increased CD4^+^ and CD8^+^T cell counts in peripheral blood (Zhang et al., 2017a). In a clinical research, Shenfu injection was used to treat elderly severe pneumonia. It could decrease the blood level of IL-6, TNF-α and IL-8, improve the vital signs of the patients, shorten the ICU hospital stays and reduce the mortality of the patients (Lv et al., 2017). Cumulative studies have shown that Shenfu injection possesses anti-inflammatory effect through multiple targets and pathways, especially inhibiting the NF-kappa B (Li et al., 2016). Ginsennoside is considered as the effective compound in Shenfu injection. Researchers found that Ginsennoside Rd could down-regulate caspase-3 expression and decrease the expression level of IL-6, TNF-α and IL-1β in a rat model of Alzheimer disease. It also has the function of anti-inflammation, anti-oxidation and anti-apoptosis (Liu et al., 2012).

Network pharmacology analyze drug effect through multiple disciplines, which provides a method to discover drugs for complex diseases (Hopkins, 2007). Shenfu injection can be used in the treatment of acute respiratory distress syndrome and acute exacerbation of chronic obstructive pulmonary disease as recommended by expert consensus. Therefore, we explored the feasibility and mechanism of Shenfu injection for the treatment of IPF using network pharmacology analysis and experiments in IPF mouse model. In this study, we performed network pharmacology analysis on the Shenfu injection in the treatment of IPF, and used protein-protein network analysis and enrichment analysis to identify hub genes and potential pathways, which may help to better understand the mechanism of Shenfu injection in the treatment of IPF. We further validated that Shenfu injection could significantly reduce pulmonary fibrosis in a mouse model.

## Materials and Methods

### Collection of Compounds and Target Genes for Shenfu Injection

Since Shenfu injection is used by intravenous injection, all of its components were analyzed. Traditional Chinese Medicine Systems Pharmacology Database and Analysis Platform (Ru et al., 2014) (TCMSP) were used to collect all compounds of Panax ginseng C.A. Mey (hongshen) and Aconitum carmichaelii Debeaux (fuzi). Because the Chinese names of hongshen and Fuzi have special latin names in database of TCMSP, we used Ginsen Radix Et Rhizoma Rubra (hongshen) and Aconiti Lateralis Radix Praeparata (Fuzi) to obtain their related target proteins (Table 1). Then the target proteins were standardized to human-related genes through the Uniprot database (UniProt Consortium, 2018).

**TABLE 1:** Information of Shenfu injection.

Latin name	English name	TCMSP name	Chinese name	Origin of place
*Panax ginseng C.A. Mey*	*Radix ginseng rubra*	*Ginsen Radix Et Rhizoma Rubra*	Hongshen	Jilin, China
*Aconitum carmichaelii Debeaux*	*Aconitine*	*Aconiti Lateralis Radix Praeparata*	Fuzi	Sichuan, China

### Screening of Idiopathic Pulmonary Fibrosis-Related Genes

We utilized “idiopathic pulmonary fibrosis” as a keyword to collect genes from the Genecards (https://www.genecards.org/) and OMIM (Hamosh et al., 2005) databases on April 2020, and chose “*Homo sapiens*” as the species option.

### Screening of Idiopathic Pulmonary Fibrosis-Related Genes Targeted by Shenfu Injection

The Venn diagram visualizing the intersection of genes between Shenfu injection and IPF was drawn by the OmicStudio tools at https://www.omicstudio.cn/tool.

### Analysis of Protein-Protein Interaction Network

The String database (Szklarczyk et al., 2019) was used to analyze the PPI network of the intersected target genes, organism was selected as “*Homo sapiens*” and the minimum required interaction score was selected as “medium confidence 0.4.” Moreover, the Cytoscape 3.7.2 software was used to visualize the PPI network.

### Enrichment Analysis of Target Genes

The Metascape platform (Zhou et al., 2019) online analytical tool was used for gene functional enrichment and Kyoto Encyclopedia of Genes and Genomes (KEGG) pathway enrichment. The hub genes were uploaded to the online tool and the significant pathways or functions were obtained based on the minimum overlap value of three and *p* value cutoff less than 0.01. Then the Cytoscape 3.7.2 software was applied to visualize the network of KEGG pathways and related target genes.

### Molecular Docking

The docking studies of the representative compounds and targets were performed with AutoDock 4.2. The BIOVIA Discovery Studio (San Diego: Dassault Systèmes) was used for pre-processing the chemical structures and biomolecules. The metal ions and/or substrate molecules (if any) were kept in the binding pocket of the targets. The Lamarckian genetic algorithm search was employed for the docking. The key residues of the binding pocket were kept flexible. The center of the binding pockets of the individual targets was selected for the grid placement. A total of 60 runs along with 2.5 million energy evaluation steps were employed. The representative pose selection was carried out based on the cluster analysis of the docked poses. The PyMOL Molecular Graphics System (Version 1.8.4.0, Schrödinger, LLC) and PLIP webserver (Salentin et al., 2015) were used for the visualizations and graphics generations.

### Shenfu Injection

Shenfu injection (batch number:18120501007) is a solution extracted from Panax ginseng C.A.Mey and Aconitum carmichaeli Debeaux. Its quality meets the standard of China Food and Drug Administation (approval No: WS3-B-3427-98-2013 and P.ZL.205-001) (Table 2). Panax ginseng C.A.Mey and Aconitum carmichaeli Debeaux were soaked and extracted to the solution respectively, then mixed together to form Shenfu injection. The specimens were stored in the herbarium of Ya’an 999 Pharmaceutical Group Co. Ltd. (Sichuan Province, China).

**TABLE 2:** Quality analysis of Shenfu injection (Batch Number: 18120501007).

Item	Content	Quality control
Finger print consistency	0.9	≥0.9
Total saponins	1.0 mg/ml	0.7–1.7 mg/ml
Ginsenoside Rg1	0.1 mg/ml	≥0.08 mg/ml
Ginsenoside Re	0.1 mg/ml	≥0.06 mg/ml

*Data were derived from product inspection report of Ya’an 999 Pharmaceutical Group Co. Ltd. Sichuan Province, China.

### Pulmonary Fibrosis Mouse Model

C57BL/6 mice (male, 25 ± 2 g, 8–9 weeks old, Vital River Laboratory Animal Technology Co., Ltd. Beijing, China) were fed with the standard diet under SPF conditions (60–70% humidity, 24 ± 1°C, 12/12 h dark-light cycle). The mice were divided into three groups (8 mice per group): control group, model group and Shenfu injection therapy group. Intratracheal instillation of bleomycin (Zhejiang HISUN Pharmaceuticals Co., Ltd.) was used at 15 USP/kg to induce pulmonary fibrosis in mice. Intraperitoneal injection of Shenfu injection (Ya’an 999 Pharmaceutical Group Co. Ltd. Sichuan Province, China) was given at 10 ml/kg once daily after bleomycin injection from day 1 to day 7. The dosage of Shenfu injection was based on a previous study (Xu et al., 2020). The animals were sacrificed on day 4 (n = 3 mice per group) or 14 (n = 5 mice per group). The left lung was used for hematoxylin-eosin staining, Masson’s trichrome stain, Sirius red staining, immunohistochemical staining and TdT-mediated dUTP nick end labeling (TUNEL) staining; and the right lung was used for Western blot. The Animal Studies Committee of China-Japan Friendship Hospital (Approval certificate number: zryhyy12-20-07-2) approved all the animal experiments in our study. All experimental procedures were performed according to the Principles of Laboratory Animal Care.

### Histological Staining and Immunostaining

The left lung of mice was dehydrated, paraffin-embedded and cut into 4-μm sections. Then the lung sections were subjected with H&E staining, Masson’s trichrome stain, Sirius red staining and immunohistochemical staining. Primary antibodies used were anti-α-SMA (ab124964, Abcam) and anti-collagen I (ab88147, Abcam).

### TUNEL Staining

The apoptotic cells in the lung tissues were detected by TUNEL staining (CF488 TUNEL Cell Apoptosis Detection Kit, Servicebio) according to the manufacturer’s instructions. Frozen lung sections (4-μm) were defrosted for 20 min at room temperature, permeabilized with 0.2% Triton X-100 for 5 min on ice, and washed twice for 5 min in PBS. Then the samples were stained with TUNEL reaction mixture for 60 min at 37°C in dark, washed twice for 5 min in PBS and mounted with Mounting Medium with DAPI (ab104139, Abcam). The slides were visualized and fluorescent images were captured with fluorescence microscope (ZEISS).

### Western Blot

The right lungs of mice were lysed and the protein concentrations were measured by BCA Protein Assay Kit (P0010, beyotime). An equivalent amount of protein samples were separated by 4–15% BeyoGel^™^ Plus Precast PAGE (Polyacrylamide gel electrophoresis) Gel (P0465S, beyotime), electrotransferred to PVDF membranes and incubated for 2 h at room temperature with primary antibodies: caspase-3 antibody (9662, Cell Signaling Technology), IL-1β antibody (31,202, Cell Signaling Technology), Phospho-NF-κB p65 antibody (3033, Cell Signaling Technology), NF-κB p65 antibody (BS1253, Bioworld) and GAPDH antibody (60004-1-Ig, Proteintech). Then, the membranes were incubated with secondary antibodies for 1 h at room temperature and exposured with the ChemiDoc XRS + System (Bio-Rad). The results were quantified using ImageJ system (1.52a).

### Quantitative Real-time PCR (qPCR)

Total RNA was extracted using the Trizol reagent according to the manufacturer’s instructions (15596026, Invitrogen), and transcribed into cDNA (11120ES60, Yeasen). qPCR was performed using SYBR Green Master Mix (11202ES08, Yeasen), and analyzed on the ABI-7500 Real-Time PCR System. PCR primer sequences were listed in Supplementary Table 1.

### Statistical Analysis

Data were presented as mean ± standard deviation (SD). Group comparisons were analyzed with one-way ANOVA after Shapiro-Wilk normality test for normality and Bartlett’s test for the homogeneity of variance. The analysis was performed by GraphPad Prism 8. *p* value <0.05 was considered statistically significant.

## Results

### Compounds and Target Genes for Shenfu Injection

We obtained 137 compounds and 119 target proteins of Shenfu injection from TCMSP after duplicates exclusion. In addition to the hydrophobic compounds, we also list the hydrophilic compounds including mainly amino acids and nucleosides identified by column-switching HILIC–RPLC-MS/MS system (Song et al., 2015) (Table 3). Since the hydrophobic compounds were considered to be the main active ingredients, we identified 88 target genes based on the 137 compounds obtained from TCMSP after standardization on the Uniprot database.

**TABLE 3:** Composite compounds of Shenfu injection.

	—	Compounds	—
Acetoacetate	Deoxyaconitine	Lactose	Tetracosane
Acetylcholine or isomer	Deoxyandrographolide	Leucine	Threonine
Aconitate	Deoxycytidine diphosphate	L-NG-monomethylarginine	Thymidine
Aconitine	Dimethyl glycine	Lysine	Thymidine isomer
Adenine	DNOP	Malate	Thymine
Adenosine	EIC	Maleamate	TMPEA
ADP	Farnesene	Maleate	trimethylamine-N-oxide
Agidol 7	Ferulate	Malonate	Tryptophan
Alanine	Ferulate isomer	Maltose	Tyrosine
Alloaromadedrene	Ferulate isomer	Mesaconitine	Tyrosine isomer
Alpha-guaiene	Folate	Methylsuccinate	UDP
Anthranilate	Fructose	Mevalonate	UNAL
Arabinose	Fructose 1,6-bisphosphate	Myristic acid	Uracil
Arachic acid	Fumarate	Neojiangyouaconitine	Uracil
Arginine	Fuzitine	Neokadsuranic acid B	Uridine
Argininosuccinate or isomer	GABA	Neoline	Valine
Ascorbate	Galactitol	Neopterin or isomer	Vetol
Asparagine	Gallate	Niacinamide	Xanthine or isomer
Aspartate	Gentisate	Nicotinate	Xylose
Asymmetric dimethylarginine, symmetric dimethylarginine or isomer	Ginsenoside R0	Nonacosane	Zoba 3 GA
Beiwutine	Ginsenoside R0_qt	Notoginsenoside R2	Zoba EG
Benzoate	Ginsenoside Rb1	Notoginsenoside R2_qt	Zoomaric acid
Benzoylaconine	Ginsenoside Re	NSC692928	α-santalene
Benzoylhypaconine	Ginsenoside Rf	0ctacosane	β-cedrene
Benzoylmesaconine	Ginsenoside Rg2	o-isoprenyl benzophenone	β-guaiene
Benzoylnapelline	Ginsenoside Rg2_qt	Ornithine	()-aromadendrene
Beta-caryophyllene	Ginsenoside Rg3	Orotate or isomer	(1S,2S)-2-isopropenyl-4-isopropylidene-1-methyl-1-vinylcyclohexane
Beta-elemene	Ginsenoside Rg3_qt	Oxaloacetate	(2R,3R,4S,5S,6R)-2-[[(3S,5R,6S,8R,9R,10R,12R,13R,14R,17S)-3,12-dihydroxy-17-[(2S)-2-hydroxy-6-methylhept-5-en-2-yl]-4,4,8,10,14-pentamethyl-2,3,5,6,7,9,11,12,13,15,16,17-dodecahydro-1h-cyclopenta [a]phenanthren-6-yl]oxy]-6-(hydroxymethyl)oxane-3,4,5-triol
Betaine	Ginsenoside Rh2	Palmitic acid	(2S,3R,4S,5S,6R)-2-[(2S)-2-[(3S,5R,8R,9R,10R,12R,13R,14R,17S)-3-[(2R,3R,4S,5S,6R)-4,5-dihydroxy-6-(hydroxymethyl)-3-[(2S,3R,4S,5S,6R)-3,4,5-trihydroxy-6-(hydroxymethyl)oxan-2-yl]oxyoxan-2-yl]oxy-12-hydroxy-4,4,8,10,14-pentamethyl-2,3,5,6,7,9,11,12,13,15,1
Beta-sitosterol	Ginsenoside Rh4	Panaxydol	(3R,5R,8R,9R,10R,12R,13R,14R,17S)-17-[(2S)-2-hydroxy-6-methylhept-5-en-2-yl]-4,4,8,10,14-pentamethyl-2,3,5,6,7,9,11,12,13,15,16,17-dodecahydro-1H-cyclopenta [a]phenanthrene-3,12-diol
Biotin	Ginsenoside Rh4_qt	Panaxynol	(3R,8S,9R,10R,13R,14S,17R)-3-hydroxy-4,4,9,13,14-pentamethyl-17-[(E,2R)-6-methyl-7-[(2R,3R,4S,5S,6R)-3,4,5-trihydroxy-6-[[(2R,3R,4S,5S,6R)-3,4,5-trihydroxy-6-(hydroxymethyl)oxan-2-yl]oxymethyl]oxan-2-yl]oxyhept-5-en-2-yl]-1,2,3,7,8,10,12,15,16,17-decahydr
Butyrate or isobutyrate	Ginsenoside Rs1	Pantothenate	(3R,9R,10R)-heptadec-1-en-4,6-diyne-3,9,10-triol
cAMP	Ginsenoside Rs1_qt	Pentadecylic acid	(3S,5R,6S,8R,9R,10R,12R,13R,14R,17S)-17-[(2R)-2-hydroxy-6-methylhept-5-en-2-yl]-4,4,8,10,14-pentamethyl-2,3,5,6,7,9,11,12,13,15,16,17-dodecahydro-1H-cyclopenta [a]phenanthrene-3,6,12-triol
Carnitine or isomer	Ginsenoside-Rb2	Phenylalanine	(3S,5R,6S,8R,9R,10R,12R,13R,14R,17S)-17-[(2S)-2-hydroxy-6-methylhept-5-en-2-yl]-4,4,8,10,14-pentamethyl-2,3,5,6,7,9,11,12,13,15,16,17-dodecahydro-1H-cyclopenta [a]phenanthrene-3,6,12-triol
Carnosifloside I_qt	Ginsenoside-Rc	Phosphoenolpyruvate	(3S,5R,8R,9R,10R,12R,13R,14R,17S)-17-[(2S)-2-hydroxy-6-methylhept-5-en-2-yl]-4,4,8,10,14-pentamethyl-2,3,5,6,7,9,11,12,13,15,16,17-dodecahydro-1H-cyclopenta [a]phenanthrene-3,12-diol
Carnosine	Ginsenoside-Rh1	Phosphotyrosine	(4aR,9aS)-2,9,9-trimethyl-5-methylene-4,4a,6,7,8,9a-hexahydro-3h-benzo [7]annulene
Cedrol	Ginsenoside-Rh1_qt	p-hydroxyphenyllactate or isomer	(6Z,10E,14E,18E)-2,6,10,15,19,23-hexamethyltetracosa-2,6,10,14,18,22-hexaene
cGMP	Globulol	p-hydroxyphenyllactic acid or isomer	(butylsulfonyl)ethynylbenzene
Chasmaconitine	Glucose	Piperonal	(R)-Norcoclaurine
Chasmanine	Glutamate	Proline	11,14-Eicosadienoic acid
Cinnamate	Glutamine	p-tert-Butylanisole	13-Deoxo-13 alpha-acetyloxy-1-deoxynortaxine I
Citrate	Glutarate	Pyridoxal-5-P	14-acetyltalatisamine
Citrulline or isomer	Glyceraldehyde	Pyridoxine or isomer	14-Deoxy-11,12-didehydroandrographolide
Clovene	Glycine	Pyrrolezanthine	1-Ethenyl-1-methyl-2-(1-methylethenyl)-4-(isopropyl)-cyclohexane
Coryneine	Guanine	Quinolinate or isomer	1-Methylhistamine
Coumaran	Guanosine	Reduced glutathione or isomer	1-Methylhistamine or isomer
Creatine	Heptacosane	Rhamnose or isomer	2,7-Dideacetyl-2,7-dibenzoyl-taxayunnanine F
Creatinine	Hetisine	Salicylate	20(R)-ginsenoside Rg2
Cysteine	Hexacosane	Salicylurate	20(S)-ginsenoside Rg3
Cytidine	Histamine or isomer	Salsolinol	20(S)-ginsenoside Rg3_qt
Cytosine	Histidine	Sanchinoside C1	20(S)-Ginsenoside-Rh1_qt
Daturic acid	Histidine isomer	Senbusine A	2-aminoadipate
Delavaconitine	Hokbusine A	Senbusine B	2-Deoxyadenosine
Delbruline	Homogentisate	Senbusine C	2-Deoxycytidine
Delbrusine	Homoserine	Serine	2-Isopropylmalate
Delcorine	Hydroxyproline	Shikimate	3,4-Dihydrobenzoate
Delphamine	Hypaconitine	Sitosterol	3,4-Dihydroxyphenylacetate
Delphin	Ignavine	Songorine	3-Oh-anthranilate or isomer
delphin_qt	Inosine	Sorbitol	3-Phosphoglycerate
Delsemine B	Inositol	Sorbitol or isomer	4-Hydroxybenzoate or isomer
Delsoline	Isoleucine	Spathulenol	4-Pyridoxic acid
Delta-amorphene	Isotalatizidine	Stockin-53073	58,924_fluka
Deltamine	Jesaconitine	Succinate	5-Hydroxytryptophan
Deltoin	Karakoline	Sucrose	5-Phenyl-4E-pentenol
Demethyldelavaine A	Karanjin	Talatizamine	6-Demethyldesoline
Demethyldelavaine B	Kynurenate	Tamarixinol	—
Denudatine	Lactate	Taurine or isomer	—

*Data were from the TCMSP database and a reference using column-switching HILIC–RPLC-MS/MS system as in the identification method (Song et al., 2015).

### Idiopathic Pulmonary Fibrosis-Related Genes

We first identified 2793 genes from the Genecards database, and then we retained 1396 genes with median target score greater than or equal to 4.86 (Target Score: maximum:124.57; minimum: 0.24; median:4.86). In addition, we obtained 322 genes from the OMIM database. Finally, a total of 1664 genes were obtained after eliminating the duplicates.

### Gene Intersection of Shenfu Injection and Idiopathic Pulmonary Fibrosis

**FIGURE 1:**
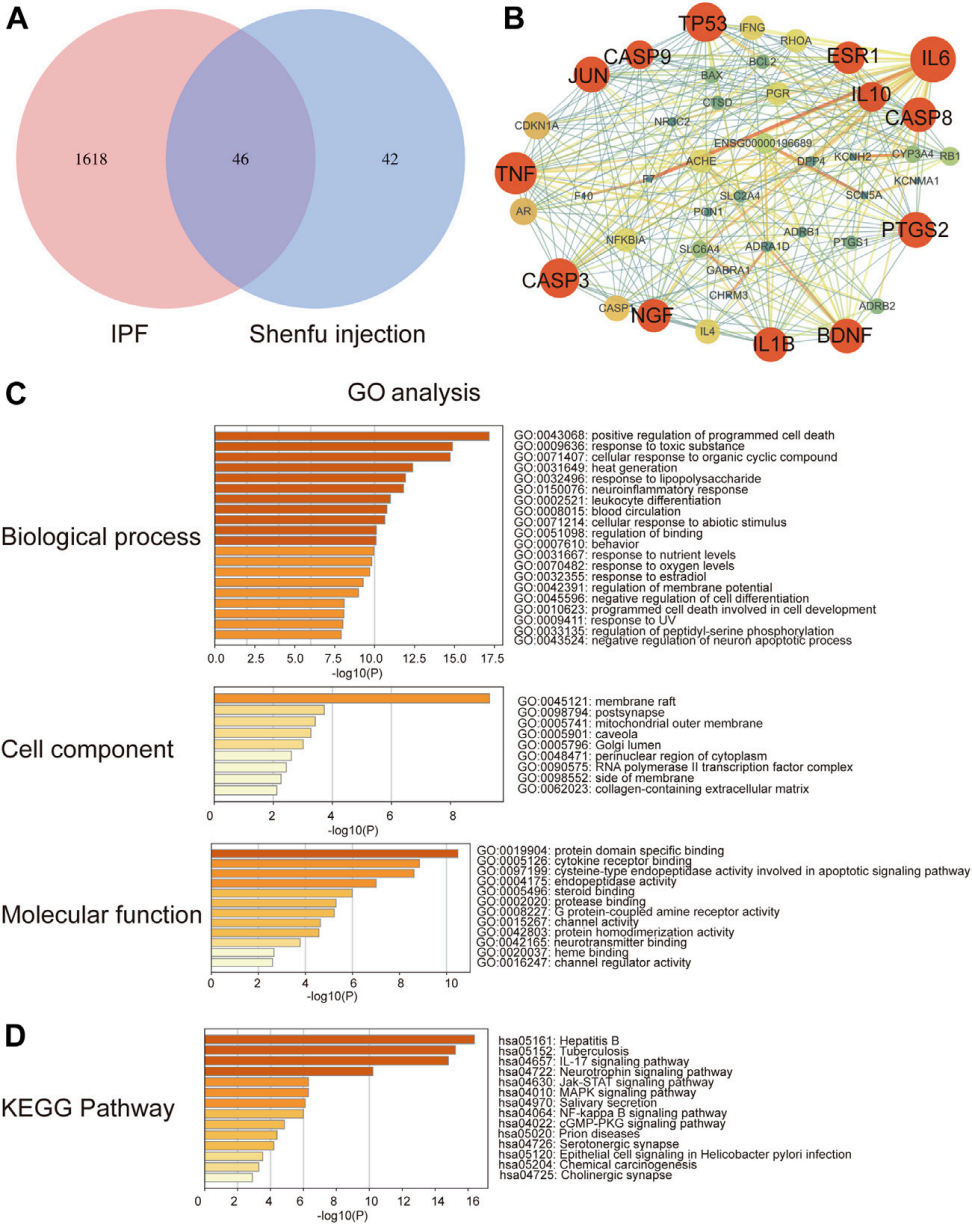
Network pharmacology analysis of Shenfu injection and IPF. **(A)** The intersection of targeted genes between Shenfu injection and IPF shown as Venn diagram. **(B)** The PPI network analysis: the node represents the protein and the edge represents the connection between proteins; the size of the node represents the importance of the gene in the network. **(C)** GO enrichment analysis of potential targets genes including biological process, cellular components and molecular function. **(D)** KEGG pathway enrichment analysis of the top 14 pathways.

In order to obtain the intersected genes between Shenfu injection and IPF, we performed a venn analysis on the target genes of Shenfu injection and IPF. As shown in Figure 1A, a total of 46 genes were identified as intersected genes of Shenfu injection and IPF.

### Protein-Protein Interaction Network Analysis of Gene Intersection

To explore the relationship of the target genes, we constructed a PPI network using the string database. The PPI network contained 46 nodes and 272 edges and the average node degree was 11.8. We further visualized the PPI network by Cytoscape 3.7.2 software (Figure 1B). The red circles represent the hub genes. The bigger the circle, the more important the gene is in the PPI network. In the PPI network, the clustering coefficient was 0.613, and the network density and centralization were 0.288 and 0.454, respectively. Considering that the higher the node degree, the more important it is in the network, we then selected the degree of node as the evaluation parameter. As a result, we screened out 13 hub genes including IL-6, TNF, TP53, CASP3, JUN, PTGS2, CASP8, IL-1B, BDNF, NGF, ESR1, IL10 and CASP9 with degrees higher than average (median degree = 16) (Table 4).

**TABLE 4:** Characterization parameters of hub genes.

Target gene	Degree	Betweenness centrality	Closeness centrality
IL6	31	0.2075	0.7544
TNF	28	0.0782	0.6825
TP53	26	0.0498	0.6615
CASP3	26	0.0475	0.6615
JUN	23	0.0185	0.6143
PTGS2	23	0.0210	0.6324
CASP8	22	0.0178	0.5811
IL1B	22	0.0344	0.6324
BDNF	22	0.0751	0.6418
NGF	21	0.0286	0.6232
ESR1	20	0.0219	0.5972
IL10	18	0.0096	0.5658
CASP9	17	0.0094	0.5584

*Data were analyzed by Cytoscape 3.7.2 software.

### Gene Ontology and Kyoto Encyclopedia of Genes and Genomes Pathway Enrichment Analysis

The GO analysis enriched 1048 biological processes, 34 cellular components and 67 molecular function items (*p* < 0.01) by using the Metascape platform (Figure 1C). Among the top five enrichments, in biological processes, genes were involved in cell death and response to stimuli, including positive regulation of programmed cell death, response to toxic substance, cellular response to organic cyclic compound, heat generation and response to lipopolysaccharide. In the cellular components, genes were enriched in the membrane-associated subcellular structures, including membrane raft, postsynapse, mitochondrial outer membrane, caveola and Golgi lumen. In the molecular functions, genes were enriched in protein domain specific binding; exogenous signal transduction pathways, including cytokine receptor binding and steroid binding; apoptosis-related functions including cysteine-type endopeptidase activity involved in apoptotic signaling pathway and endopeptidase activity.

Moreover, we performed KEGG enrichment analysis and found that the hub genes were mainly involved in infection and inflammation pathways. The top five enrichments include hpatitis B, tuberculosis, IL-17 signaling pathway, JAK-STAT signaling pathway and neurotrophin signaling pathway (Figure 1D).

### Network of Target Genes and Pathways

**FIGURE 2:**
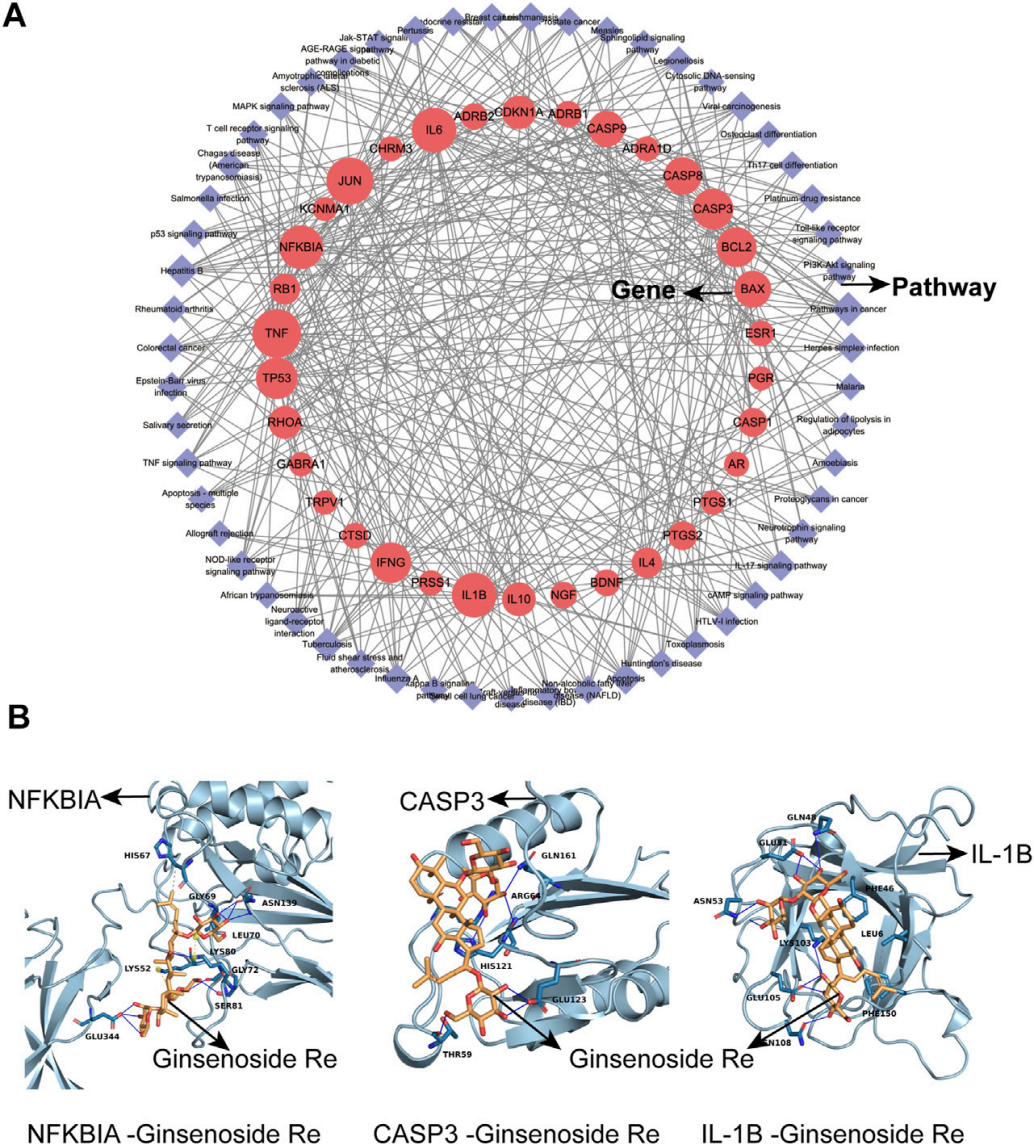
The network of target gene-pathway interactions and molecular docking analysis. **(A)** The network of target gene-pathway interactions. Red circle stands for target genes and purple diamond stands for pathways. The size of the red circle represents the importance of the target gene in this network. **(B)** Molecular docking analysis. 3D visualization of NFKBIA-Ginsenoside Re, CASP3-Ginsenoside Re and IL-1B-Ginsenoside Re interactions are presented.

After obtaining the pathways from the Metascape platform, we further used Cytoscape 3.7.2 software to visualize the relationships between the pathways and genes. As shown in Figure 2A, the red circles represent the target genes and the purple diamonds represent the pathways. There were 89 nodes in the network (55 pathways, 34 target genes), and the median of genes degree was 16.5. Among them, target genes with degrees higher than average were TNF, JUN, IL-6, IL-1B, NFKBIA, TP53, CASP3, IFNG, BCL2, CASP8 and BAX (Table 5), which may be important targets for Shenfu injection in the treatment of IPF. The median of pathways degree was nine. Pathways with degrees higher than average included pathways in cancer, infection/inflammation including tuberculosis, hepatitis B and IL-17 signaling pathway; and apoptosis (Table 6).

**TABLE 5:** Target gene network and node characterization parameters.

Target gene	Degree	Betweenness centrality	Closeness centrality
TNF	32	0.1041	0.4607
JUN	30	0.1537	0.4809
IL6	27	0.0689	0.4334
IL-1B	27	0.0571	0.4251
NFKBIA	26	0.1079	0.4512
TP53	23	0.0572	0.4055
CASP3	22	0.0426	0.3982
IFNG	22	0.0415	0.4055
BCL2	21	0.0440	0.3946
CASP8	18	0.0260	0.3810
BAX	17	0.0221	0.3777

*Data were analyzed by Cytoscape 3.7.2 software.

**TABLE 6:** KEGG Pathway network and node characterization parameters.

Pathway	Degree	Betweenness centrality	Closeness centrality
Pathways in cancer	14	0.0498	0.4444
Tuberculosis	12	0.0247	0.4231
Hepatitis B	12	0.0182	0.4314
Apoptosis	11	0.0243	0.4272
IL-17 signaling pathway	10	0.0208	0.4314

*Data were analyzed by Cytoscape 3.7.2 software.

### Molecular Docking of Active Compounds and Targeted Proteins

We used Cytoscape 3.7.2 software to analyze active compounds (Table 7). We selected compounds including beta-sitosterol, beta-elemene, myristic acid, TMPEA and the main content Ginsenoside Re as active compounds targeting TNF, IL-6, IL-1B, CASP3 and NFKBIA proteins. Generally, the negative value of the docking score means that they can bind to each other, and the positive value means that they cannot bind. The smaller the score value, the stronger the binding force is. Molecular docking results showed that these five compounds could bind to the target proteins. The docking score of Ginsenoside Re- NFKBIA was −9.046 kcal/mol, Ginsenoside Re- CASP3 was −9.273 kcal/mol, and Ginsenoside Re- IL-1B was −7.806 kcal/mol, showing that Ginsenoside Re had a relatively strong affinity with NFKBIA, CASP3 and IL-1B (Figure 2B). These results indicated that Shenfu injection may be functional in the treatment of IPF by targeting NFKBIA, CASP3 and IL-1B by its main content Ginsenoside Re. The docking details were recorded in Table 8 and Supplementary Table 2.

**TABLE 7:** Main active compounds characterization parameters.

Pathway	Degree	MOL ID	Betweenness centrality	Closeness centrality
Beta-sitosterol	38	MOL000358	0.1951	0.4669
Beta-elemene	26	MOL000908	0.1311	0.4273
Myristic acid	24	MOL001393	0.0691	0.4075
TMPEA	19	MOL002399	0.0529	0.4099

*Data were analyzed by Cytoscape 3.7.2 software.

**TABLE 8:** Molecular docking score (kcal/mol).

Gene	PDB	Molecules				
		MOL000358	MOL000908	MOL001393	MOL002399	MOL005338
NFKBIA	1SVC	−1.962	−0.309	−1.315	−1.826	−9.046
TNF	6X81	No binding	−0.946	1.245	−3.494	No binding
IL-6	1ALU	No binding	−0.853	−1.382	−1.967	No binding
IL1B	1ITB	−1.440	−0.682	−0.449	−2.405	−7.806
CASP3	2XYP	−2.493	−1.406	−0.923	−3.653	−9.273

### Shenfu Injection Reduces BLM-Induced Pulmonary Fibrosis in Mice

**FIGURE 3:**
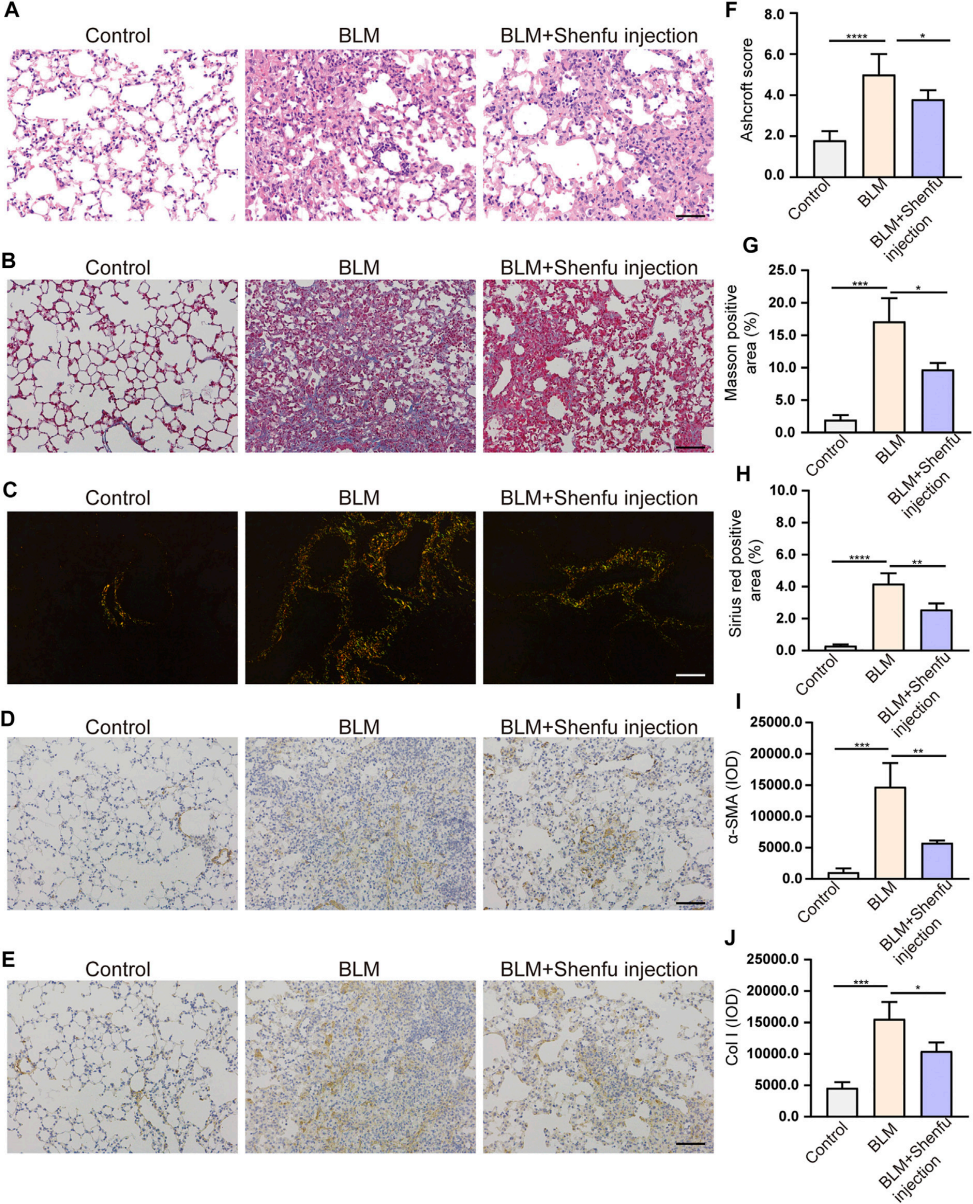
Shenfu injection alleviates pulmonary fibrosis. Representative photomicrographs of H&E staining **(A)** (*n* = 5), Masson’s trichrome staining **(B)** (*n* = 3) and Sirius red staining **(C)** (*n* = 3). Representative immunohistochemistry photomicrographs of α-SMA **(D)** (*n* = 3) and collagen I **(E)** (*n* = 3). Quantification of pulmonary fibrosis by Ashcroft score **(F)**, Masson positive area **(G)**, Sirius red positive area **(H)**, α-SMA positive signals **(I)** and collagen I positive signals **(J)**. Scale bar: 100 μm. The results are expressed as mean ± SD. ****p < 0.0001; ***p < 0.001; **p < 0.01; *p < 0.05. IOD, integrated optical density.

We established bleomycin-induced pulmonary fibrosis on a mouse model. H&E staining of lung sections showed that bleomycin induced destruction of alveolar structures in mice, and the alveolar space was filled with fibrous tissue (Figure 3A). Shenfu injection-treated group showed lower score than bleomycin-treated group as assessed by Ashcroft scoring (*p* < 0.05, Figure 3F). Masson’s trichrome staining showed the deposition of extensive collagen fibers in the lung interstitium (Figure 3B). Sirius red staining showed birefringent red collagen I and green collagen III under polarized light microscopy (Figure 3C). Shenfu injection treatment remarkably ameliorated these pathological injuries and alleviated collagen deposition (Figure 3G,H). The expression of α-SMA and collagen I was analyzed by immunohistochemical staining on the lung sections. Our data showed that the expression of α-SMA and collagen I increased in bleomycin-treated group, and Shenfu injection treatment significantly inhibited the expression of α-SMA and collagen I in the lung sections compared with bleomycin-treated group (Figure 3D,E,I,J).

### Shenfu Injection Reduces Pulmonary Fibrosis by Inhibiting Inflammation and Apoptosis

**FIGURE 4:**
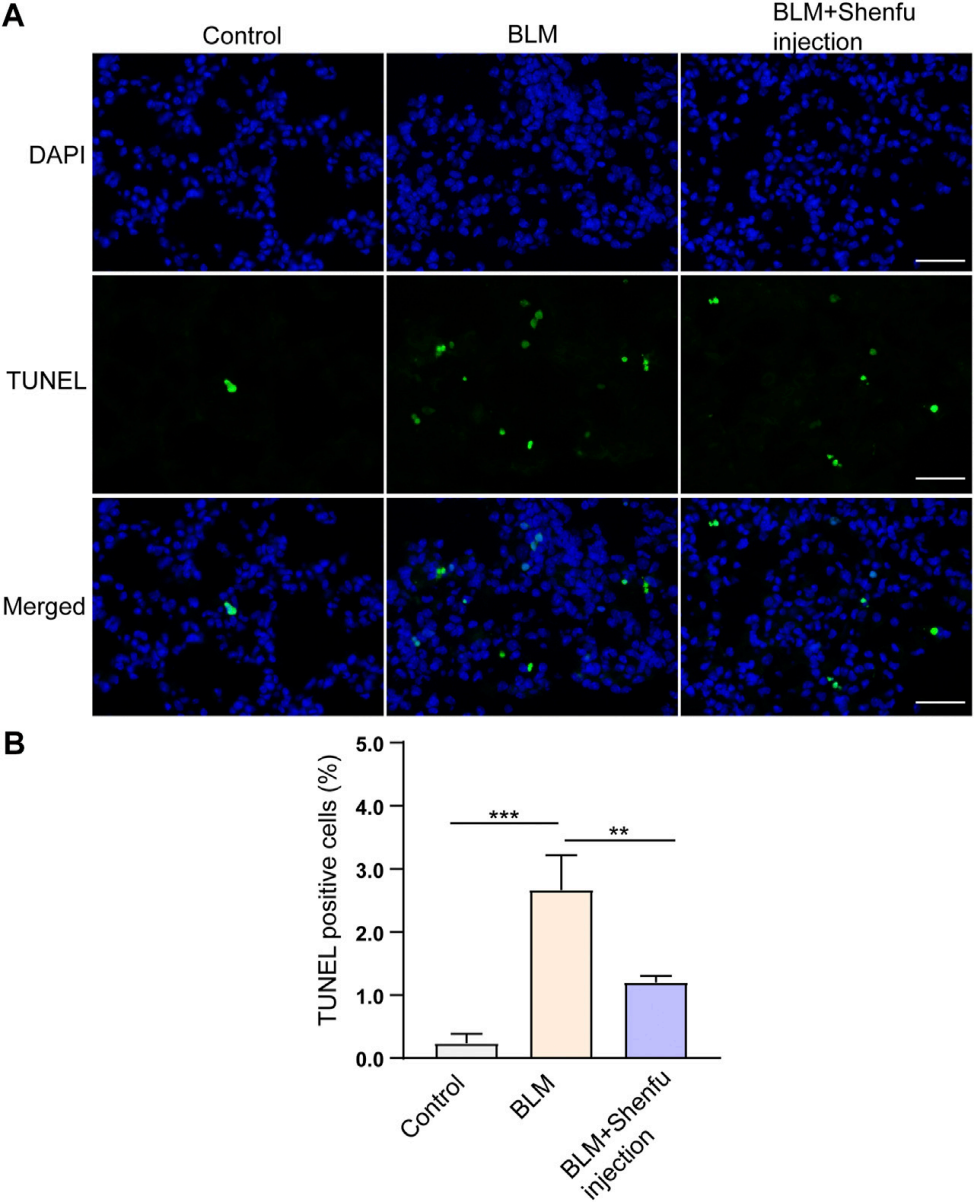
Shenfu injection suppresses BLM-induced apoptosis. **(A)** Representative TUNEL staining using lung tissues 4 days after BLM treatment. **(B)** Quantification of TUNEL positive cells. Scale bar: 50 μm. The results are expressed as mean ± SD (*n* = 3 mice per group). ***p < 0.001, **p < 0.01.

**FIGURE 5:**
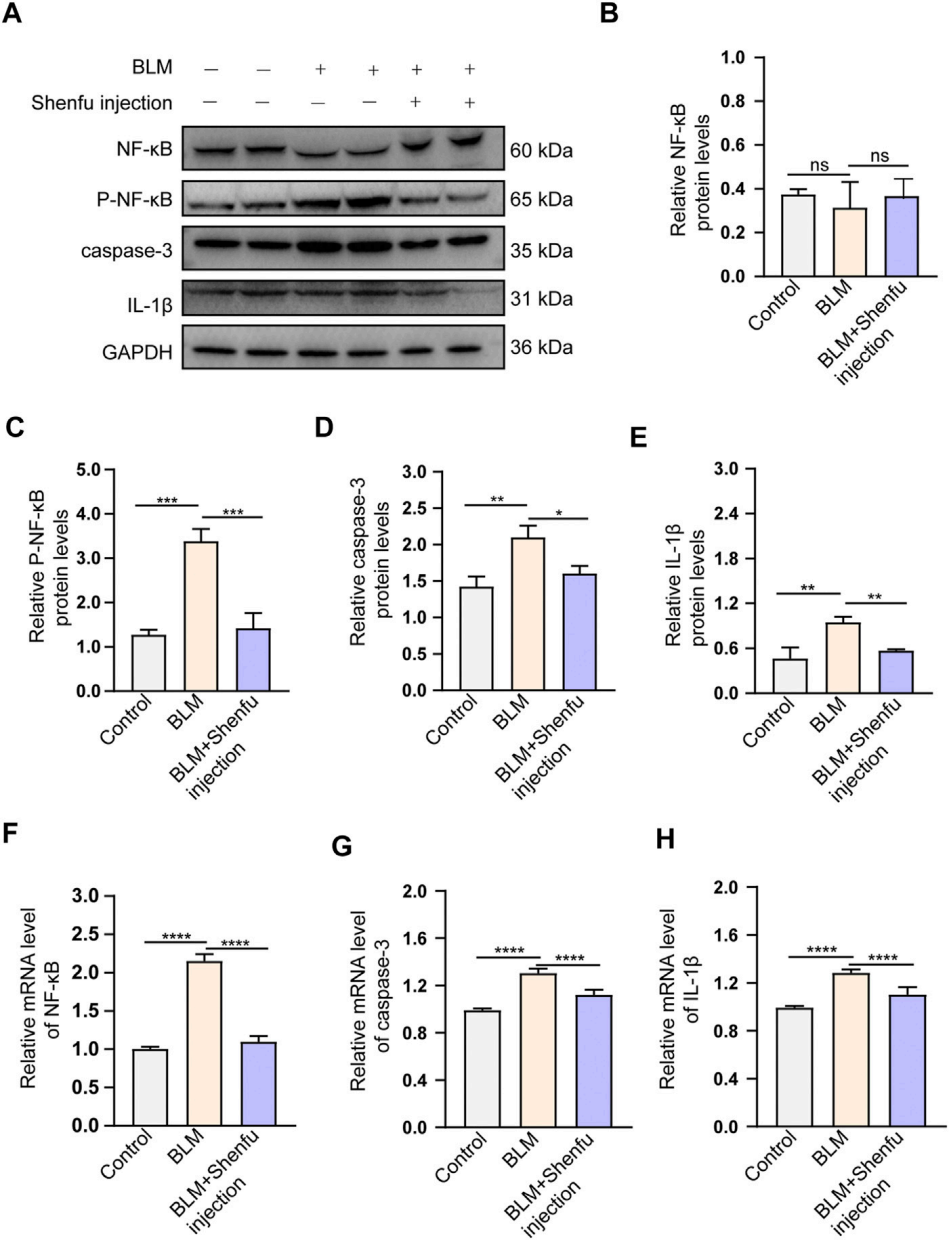
Shenfu injection inhibits BLM-induced pulmonary fibrosis by targeting NFKBIA, CASP3 and IL-1B. **(A)** Western bot analysis of total NF-κB, Phospho-NF-κB, caspase-3 and IL-1β in each group. **(B–E)** Quantitative analysis of total NF-κB **(B)**, Phospho-NF-κB **(C)**, caspase-3 **(D)** and IL-1β **(E)** protein levels in each group (*n* = 3 mice per group). **(F–H)** Quantification of relative mRNA levels of NF-κB **(F)**, caspase-3 **(G)** and IL-1β **(H)** in each group (*n* = 3 mice per group). Data are presented as mean ± SD. ****p < 0.0001; ***p < 0.001; **p < 0.01; *p < 0.05.

To verify the mechanisms of Shenfu injection in the treatment of pulmonary fibrosis proposed by network pharmacology analysis, we performed TUNEL staining to observe the apoptotic cells in the lung sections, qPCR to examine the mRNA level of NFKBIA, CASP3, and IL-1B, and Western blot to examine the expression levels of inflammation-related proteins, including caspase-3, IL-1β, phosphorylated NF-κB and total NF-κB in the lung tissues from the three groups. The results showed that the number of TUNEL-positive cells increased in BLM-treated group compared with the control, and Shenfu injection significantly reduced the number of TUNEL-positive cells induced by BLM treatment. (Figure 4). The mRNA and protein level of caspase-3 was also upregulated in BLM-treated group, and could be reversed in Shenfu injection-treated group (*p* < 0.05, Figure 5A,D,G). These results indicated that BLM treatment could induce apoptosis in pulmonary cells, and Shenfu injection inhibited the apoptosis process, which may be one of its mechanisms to alleviate subsequent pulmonary fibrosis.

For the regulation of inflammatory process, we found that BLM treatment upregulated the protein and mRNA level of IL-1β, and Shenfu injection treatment significantly reduced the increased expression of IL-1β (*p* < 0.05, Figure 5A,E,H). In addition, BLM treatment upregulated mRNA level of NF-κB, and Shenfu injection treatment significantly reduced the mRNA level. Interestingly, the total protein level of NF-κB did not changed significantly. Since the phosphorylated NF-κB is the active form of this protein, we detected the protein level of phosphorylated NF-κB. It increased in BLM-treated group, and this increasement was significantly inhibited by Shenfu injection (Figure 5A–C,F), indicating that instead of affecting the total protein amount of NF-κB, Shenfu injection may function through inhibiting its phosphorylation. The above results together demonstrated that inhibition of inflammatory response is another mechanism of Shenfu in the treatment of pulmonary fibrosis.

## Discussion

Shenfu injection is a pure Chinese patent medicine modified from the classical Chinese medicine “Shenfu decoction” and extracted from ginseng and Fuzi by using the modern pharmaceutical technology. As “Extension of Famous Medical Prescriptions” records: “There is nothing as good as ginseng to nourish acquired Qi, and there is nothing as good as Fuzi to replenish innate Qi,” Shenfu injection is mainly used in infectious diseases, haemorrhage, hemorrhagic shock and other Yang Qi burst off Syndromes in clinics. It is also applied in the Qi deficiency Syndrome, such as cough, shock, panic, stomachache, diarrhea and so on. In traditional Chinese medicine, IPF is considered to be in the category of pulmonary arthralgia and cough, and its root is Qi deficiency of the lung and spleen. Therefore, Shenfu injection might have some effects on IPF based on the theory of traditional Chinese medicine. Based on the studies with modern medical research methods, the therapeutic mechanism of Shenfu injection could be attributed to the promotion of cell repair and inhibition of cell apoptosis, as well as inhibition of inflammation, oxidation, ischemia and hypoxia (Liu et al., 2012; Lv et al., 2017; Chen et al., 2020). For instance, a recent study found that Shenfu injection inhibited TLR4/NF-κB signaling pathway, reduced systemic inflammatory response and played a protective role in the post-resuscitation myocardial dysfunction (Gu et al., 2021). Apoptosis of alveolar epithelial cells and the abnormal repair of alveolar injury is considered to be the pathological basis of IPF, while inflammation plays an important role in the induction and acute exacerbation of IPF. These characteristics are also consistent with the therapeutic mechanism of the Shenfu injection.

In this study, network pharmacological analysis revealed 46 genes correlated with Shenfu injection and IPF. The results indicated that Shenfu injection has a potential therapeutic effect on IPF, and its curative effect may be exerted through anti-inflammation and anti-cell apoptosis, involving pathways in tuberculosis, hepatitis B, apoptosis and IL-17 signaling.

As IL-6 and TNF co-stimulate MIP1-α expression of alveolar macrophages in bleomycin-induced mice, researchers proposed that IL-6 and TNF are part of the cytokine network in bleomycin-induced pulmonary pathophysiology (Smith et al., 1998). IL-6 and TNF-α polymorphism analysis in IPF patients showed that IL-6 and TNF-α genes differed between the IPF and normal population and both related to the progression of IPF (Pantelidis et al., 2001). In lung tissue specimens, TNF-α and IL-1β were overexpressed in acute pulmonary fibrosis while presented a low expression level in old IPF patients, suggesting that TNF-α and IL-1β may be involved in the initiation of pulmonary fibrosis. Besides, the serum and BALF level of IL-1β is higher in IPF patients than healthy controls (Pan et al., 1996; Barlo et al., 2011). Shenfu injection alleviated lung injury in a rat systemic inflammatory response syndrome model by inhibiting NF-B activation and decreasing the plasma level of IL-6 and TNF-α (Wang et al., 2008). In a vascular endothelial cell damaging model, Shenfu injection cloud increase the level of Bcl2 protein and reduce caspase-3 and Bax levels, demonstrating that Shenfu injection has the function of anti-apoptosis and anti-oxidance (Hong et al., 2015). The molecular docking results showed that compounds of Shenfu injection could bind to these targets, especially its main content Ginsenoside Re had strong affinity with NFKBIA, CASP3 and IL-1B. Our results showed that Shenfu injection may interfere with inflammatory response and cell apoptosis by targeting NFKBIA, CASP3 and IL-1B.

IPF has the common photomechanics of epithelial damage, abnormal repairment and epithelial-mesenchymal transition with lung cancer, and it is considered to be associated with lung cancer. Researchers observed TP53 status in surgical resection of lung cancer in IPF patients, and found that the peripheral parts of fibrotic zone had high mutation rate of TP53 gene. Another research found that lung cancer with interstitial lung diseases had high risk for pleural invasion (Kawasaki et al., 2001; Hata et al., 2017). Moreover, a meta-analysis evaluated the function of Shenfu injection by objective tumor response, disease control rate, karnofsky performance score, adverse effects and indicators of cellular immune function, and the result indicated that Shenfu injection could improve immune function of lung cancer patients and reduce adverse effect of chemotherapy (Cao et al., 2017). The interaction mechanism between Shenfu injection and lung cancer are still unclear. A cohort study found that IPF has a high risk of lung cancer. After adjusting the risk of smoking, the rate ratio (RR) for the incidence of IPF with lung cancer was 8.25 (890 IPF patients, 5884 control participants, 95% CI 4.70–11.48) (Hubbard et al., 2000). Our results showed that Shenfu injection may exert anti-tumor function by regulating P53 and JUN, which may add beneficial effects to the treatment of IPF.

Notably, infection, inflammation and apoptosis participate in the process of IPF. Viral and bacterial infections play a role in the process of IPF by causing alveolar injury and apoptosis. A meta-analysis from 20 studies (634 IPF patients, 653 controls) analyzed 19 virus species, demonstrating that the incidence of IPF increased by the infection of Epstein-Barr virus (EBV), cytomegalovirus (CMV), human herpesvirus 7 (HHV7) and human herpesvirus 8 (HHV8) (Sheng et al., 2020). Tuberculosis and hepatitis B are a global health problem, especially in China. Tuberculosis induces pulmonary fibrosis *via* macrophage apoptosis, recruiting TNF-α, TGF-β, Th1 and Th2 cytokines. Chronic hepatitis B causes defective T cell to affect the immune system and accelerates the progress of live fibrosis (Dheda et al., 2005; Nebbia et al., 2012). Besides, researchers found that B cell activating factor (BAFF, one member of the TNF family) played a key role in bleomycin-induced lung fibrosis model by regulating the IL-1β and IL-17 pathway, and the expression of BAFF increased in BALF of IPF patients (François et al., 2015). Our results showed that Shenhua injection may interfere with tuberculosis, hepatitis B, apoptosis and IL-17 signaling to treat IPF.

**FIGURE 6:**
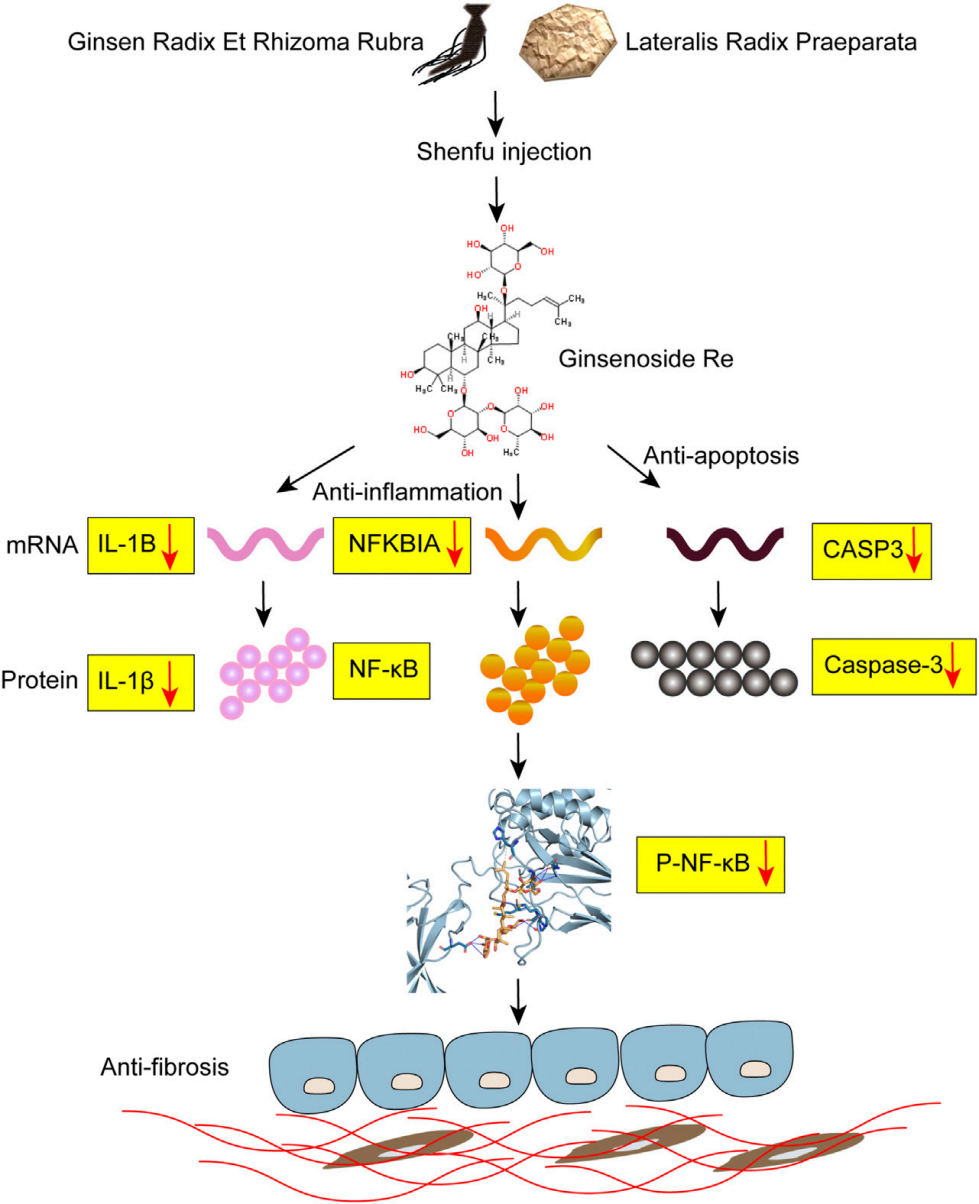
Potential mechanism of Shenfu injection alleviating BLM-induced pulmonary fibrosis. Shenfu injection may have the function of anti-inflammation and anti-apoptosis by targeting NFKBIA, CASP3 and IL-1B, and alleviate BLM-induced pulmonary fibrosis.

Finally, to examine the effect of Shenfu injection *in vivo*, we used bleomycin-induced pulmonary fibrosis mouse model and applied Shenfu injection as daily treatment. Our data showed that Shenfu injection ameliorated the expression of α-SMA and collagen I, and remarkably reduced pulmonary fibrosis. We further validated that Shenfu injection inhibited the mRNA level of NFKBIA, CASP3, and IL-1B, it also inhibited NF-κB activation and reduced the protein expression levels of caspase-3 and IL-1β. Interestingly, our WB result of total protein NF-κB did not change significantly in the BLM-treated group and the Shenfu injection-treated group. Since the mRNA level may not always be consistent with the protein level, we considered that the total protein level of NFKBIA may be affected by other post-transcriptional modifications. At the same time, we found that Ginsenoside Re—the main component of Shenfu injection—could bind to NFKBIA and inhibit its phosphorylation. Therefore, we proposed a new mechanism from network pharmacology and our experiment that Shenfu injection may influence the activity of NFKBIA by inhibiting its phosphorylation by binding it with Ginsenoside Re, instead of affecting the total amount of NFKBIA protein. Inflammation plays an important role in IPF pathogenesis, and NF-κB coordinate with the expression of inflammation genes, that contribute to pulmonary fibrosis (Li and Verma, 2002). Besides, in human IPF and BLM-induced animal lung samples, excess alveolar epithelial cell apoptosis promotes pulmonary fibrosis (Hosseinzadeh et al., 2018). Our results validated the effectiveness of Shenfu injection in the treatment of IPF, and verified its mechanism as inhibiting inflammation and apoptosis as proposed by targeting NFKBIA, CASP3, and IL-1B (Figure 6). Currently, the interaction of cancer-related pathways and IPF is not clear. How Shenfu injection ameliorates pulmonary fibrosis by regulating cancer-related genes such as JUN and TP53 is worth study in the future.

## Conclusion

According to network pharmacology-based analysis and animal experiment, we discovered that Shenfu injection has the therapeutic potential for IPF and provided possible mechanisms underlined. This is the first evidence for the feasibility of Shenfu injection in the treatment of IPF.

## Data Availability

The original contributions presented in the study are included in the article/Supplementary Material, further inquiries can be directed to the corresponding authors.

## Abbreviations

GO, gene ontology; IPF, idiopathic pulmonary fibrosis; KEGG, kyoto encyclopedia of genes and genomes; PPI, protein-protein interaction; TCMSP, traditional chinese medicine systems pharmacology database and analysis platform.
