# Supplementary material for: Feasibility and Mechanism Analysis of Shenfu Injection in the Treatment of Idiopathic Pulmonary Fibrosis
Source: Front Pharmacol. 2021 Jul 28;12:670146. doi: 10.3389/fphar.2021.670146 (PMC8356043; doi:10.3389/fphar.2021.670146)
Supplement: Supplementary file 1 [file Table1.docx]

**Table S1. Primer sequences**

| **Primer** | **Sequence 5' to 3'** |
| --- | --- |
| GAPDH-F | AGGTCGGTGTGAACGGATTTG |
| GAPDH-R | TGTAGACCATGTAGTTGAGGTCA |
| Caspase-3-F | TGGTGATGAAGGGGTCATTTATG |
| Caspase-3-R | TTCGGCTTTCCAGTCAGACTC |
| NF-κB-F | GGAGGCATGTTCGGTAGTGG |
| NF-κB-R | CCCTGCGTTGGATTTCGTG |
| IL-1β-F | GCAACTGTTCCTGAACTCAACT |
| IL-1β-R | ATCTTTTGGGGTCCGTCAACT |
