# Supplementary material for: Feasibility and Mechanism Analysis of Shenfu Injection in the Treatment of Idiopathic Pulmonary Fibrosis
Source: Front Pharmacol. 2021 Jul 28;12:670146. doi: 10.3389/fphar.2021.670146 (PMC8356043; doi:10.3389/fphar.2021.670146)
Supplement: Supplementary file 2 [file Table2.docx]

**Table S2.** **Targets and molecular docking parameters**

| **Target** | **Gene Name** | **PDB ID** | **GRID SIZE** |
| --- | --- | --- | --- |
| NF-kappa-B inhibitor alpha | NFKBIA | 1SVC | 80 Å✕80 Å✕80 Å |
| Tumor necrosis factor | TNF | 6X81 | 80 Å✕80 Å✕80 Å |
| Interleukin 6 | IL-6 | 1ALU | 80 Å✕80 Å✕80 Å |
| Interleukin-1 beta | IL1B | 1ITB | 80 Å✕80 Å✕80 Å |
| Caspase 3 | CASP3 | 2XYP | 80 Å✕80 Å✕80 Å |
